# Oxidative stress indexes as biomarkers of the severity in COVID-19 patients

**DOI:** 10.7150/ijms.102879

**Published:** 2024-11-11

**Authors:** Xin Liu, Ruohong Chen, Binghui Li, Jialiang Zhang, Peiting Liu, Bingchu Li, Fengfan Li, Weilin Zhang, Xing Lyu, Min Hu

**Affiliations:** 1 Department of Laboratory Medicine, The Second Xiangya Hospital, Central South University, Changsha, Hunan 410011, China.; 2 Center for Clinical Molecular Diagnostics, The Second Xiangya Hospital, Central South University, Changsha, Hunan 410011, China.

**Keywords:** COVID-19, oxidative stress, oxidized low-density lipoprotein, 3-nitrotyrosine

## Abstract

**Background**: SARS-CoV-2 causes a global pandemic, with severe and critically ill COVID-19 patients often experiencing poor prognoses. Severe infection with SARS-CoV-2 is associated with oxidative stress (OS) and inflammation. Detecting markers of macromolecular damage caused by OS may provide valuable insights into disease progression.

**Methods**: This study included 187 patients with laboratory-confirmed SARS-CoV-2 infection, categorized into non-severe, severe, and critically ill COVID-19 groups. We monitored the changes in serum indexes such as oxidized low-density lipoprotein (OxLDL), OxLDL/LDL-C ratio, advanced oxidation protein products (AOPP), 3-nitrotyrosine (3-NT), 8-hydroxydeoxyguanosine (8-OHdG), lipoprotein-associated phospholipase A_2_ (Lp-PLA_2_) and thromboxane B_2_ (TXB_2_) in patients with different clinical types.

**Results**: 48 non-severe patients, 90 severe patients, and 49 critically ill patients were enrolled. Compared with the non-severe group, OxLDL level and OxLDL/LDL-C ratio were increased in severe COVID-19 patients and critically ill COVID-19 patients, while 3-NT and TXB_2_ concentrations were lower in critically ill COVID-19 patients. Critically ill COVID-19 patients also exhibited lower concentrations of Lp-PLA_2_ and a higher OxLDL/LDL-C ratio compared to severe COVID-19 patients. No significant differences were observed in AOPP and 8-OHdG concentrations. Spearman's correlation analysis revealed that CRP was associated with OxLDL, OxLDL/LDL-C ratio, AOPP, 3-NT, TXB_2_, and Lp-PLA_2_ (*P* <0.05). OxLDL was identified as an independent risk factor for progression from non-severe to severe/critically ill COVID-19. OxLDL and OxLDL/LDL-C ratio demonstrated good discriminatory value between non-severe and severe/critically ill COVID-19, with the OxLDL/LDL-C ratio also distinguishing between severe and critically ill patients.

**Conclusion**: Patients with severe and critically ill COVID-19 exhibit elevated levels of oxidative damage to lipoproteins. OxLDL and the OxLDL/LDL-C ratio can serve as biomarkers for assessing disease severity in COVID-19 patients.

## Introduction

COVID-19, caused by severe acute respiratory syndrome coronavirus 2 (SARS-CoV-2), has led to a global pandemic, posing a threat to global health. The World Health Organization (WHO) has reported over 775 million confirmed cases and more than 7 million deaths worldwide since the beginning of the pandemic 1. Despite ongoing efforts, new infections and fatalities continue to occur weekly. The SARS-CoV-2 infection causes different clinical manifestations, ranging from asymptomatic infections to critical illnesses. The case fatality rate for severe COVID-19 patients hospitalized in intensive care units is approximately 40% 2. Therefore, monitoring the severity of COVID-19 is essential. However, etiological and serological methods for diagnosing SARS-CoV-2 infection cannot distinguish the severity of the infection. Serum biomarkers seem to have greater potential in this regard, but serological biomarkers for disease severity are still lacking.

There is substantial evidence that oxidative stress (OS) and inflammation play pivotal roles in the severe infections caused by SARS-CoV-2 3-5. OS refers to the imbalance between antioxidant capacity and reactive oxygen species (ROS) 6. In conjunction with cytokine storms, OS contributes to the COVID-19 pathogenesis by inducing endothelial cell dysfunction as well as the activation of the clotting cascade, leading to platelet activation, blood clotting, and microvascular thrombosis, which are key pathological mechanisms in severe cases 7-9. Inflammatory markers have been included in the guidelines as important early warning indicators of severe COVID-19 infection 10. Elevated concentrations of multiple pro-inflammatory markers, such as interleukin-6 (IL-6) and C-reactive protein (CRP), have been found in patients with COVID-19 and can be used as predictors of disease severity 11, 12. Previous reports have found that the median levels of CRP and IL-6 in patients infected with COVID-19 are 5.56 mg/L and 9.52 pg/mL, respectively. However, healthy controls with no statistically significant difference in age or gender had CRP levels of 0.40 mg/L and IL-6 levels of 4.79 pg/mL 11. Given that oxidative stress often coexists with inflammation, we speculate that some OS indicators may also have a role in predicting the severity of COVID-19 disease. There is a growing body of literature that highlights the significance of OS in COVID-19. In a severe case of COVID-19, there was increased OS in the lungs, mainly manifested by the occurrence of lipid peroxidation and oxidative DNA damage 13. However, studies on the serum markers of OS in SARS-CoV-2 infection are not comprehensive. In addition, not all participants were infected with SARS-CoV-2 for the first time at the time of enrollment. Therefore, further research is still needed.

The detection of OS-induced macromolecular damage has become a routine means to assess OS levels, including lipid, protein, and DNA 14. Therefore, in this article, markers of oxidative damage of macromolecular substances in patients with COVID-19 of different severity were mainly discussed from the perspectives of proteins, lipids, and DNA. OS converts low-density lipoprotein (LDL) to oxidized low-density lipoprotein (OxLDL), therefore, OxLDL and OxLDL/LDL-C ratio are often used to reflect the oxidation of lipoprotein 15. 3-Nitrotyrosine (3-NT) and advanced oxidation protein products (AOPP) are commonly used indicators of the oxidative damage levels of proteins 16. As for markers of DNA damage, 8-hydroxydeoxyguanosine (8-OHdG) is the most frequently represented indicator 17. OS will cause endothelial cell dysfunction and platelet activation, as well as the occurrence of coagulation cascades. Therefore, lipoprotein-associated phospholipase A_2_ (Lp-PLA_2_), an indicator of vascular inflammation, and thromboxane B_2_ (TXB_2_), an indicator of platelet activation and thrombosis, were also included as supplementary serological indicators in this study 18, 19. Additionally, CRP is used to reflect inflammation levels. We examine the concentrations of these indicators in COVID-19 patients to explore whether markers of OS can be used as biomarkers of disease severity.

## Methods

### Study population

A total of 187 patients with laboratory-confirmed SARS-CoV-2 infection from the Second Xiangya Hospital of Central South University from December 2022 to July 2023 were recruited in this study. All patients were confirmed with reverse transcriptase polymerase chain reaction (RT-PCR) or antigen test in a nasopharyngeal swab. And all patients were newly infected.

Inclusion criteria: COVID-19 patients infected with the SARS-CoV-2 were classified according to disease severity. According to the Diagnosis and treatment protocol for COVID-19 patients (Tentative 10th Version) 10, the subjects were clinically classified into mild, moderate, severe, and critically ill patients. According to previous studies, we divided the patients with mild and moderate infections into the non-severe group 20. Subjects with the following diseases or conditions were excluded from this study: (1) Patients with lung malignant tumors; (2) Patients with incomplete or missing data; (3) Children, pregnant women, and breast-feeding women.

The basic demographic parameters, previous history, medical history, clinical diagnosis, and treatment information of all subjects were obtained from the medical record system and confirmed by the study physicians. For all participants, 3 ml of serum samples were collected in a fasting state. Serum samples were stored at 4 ℃ for a short time and then stored in the -80 ℃ refrigerator immediately after packaging, and repeated freeze-thaw was avoided.

**Figure 1:**
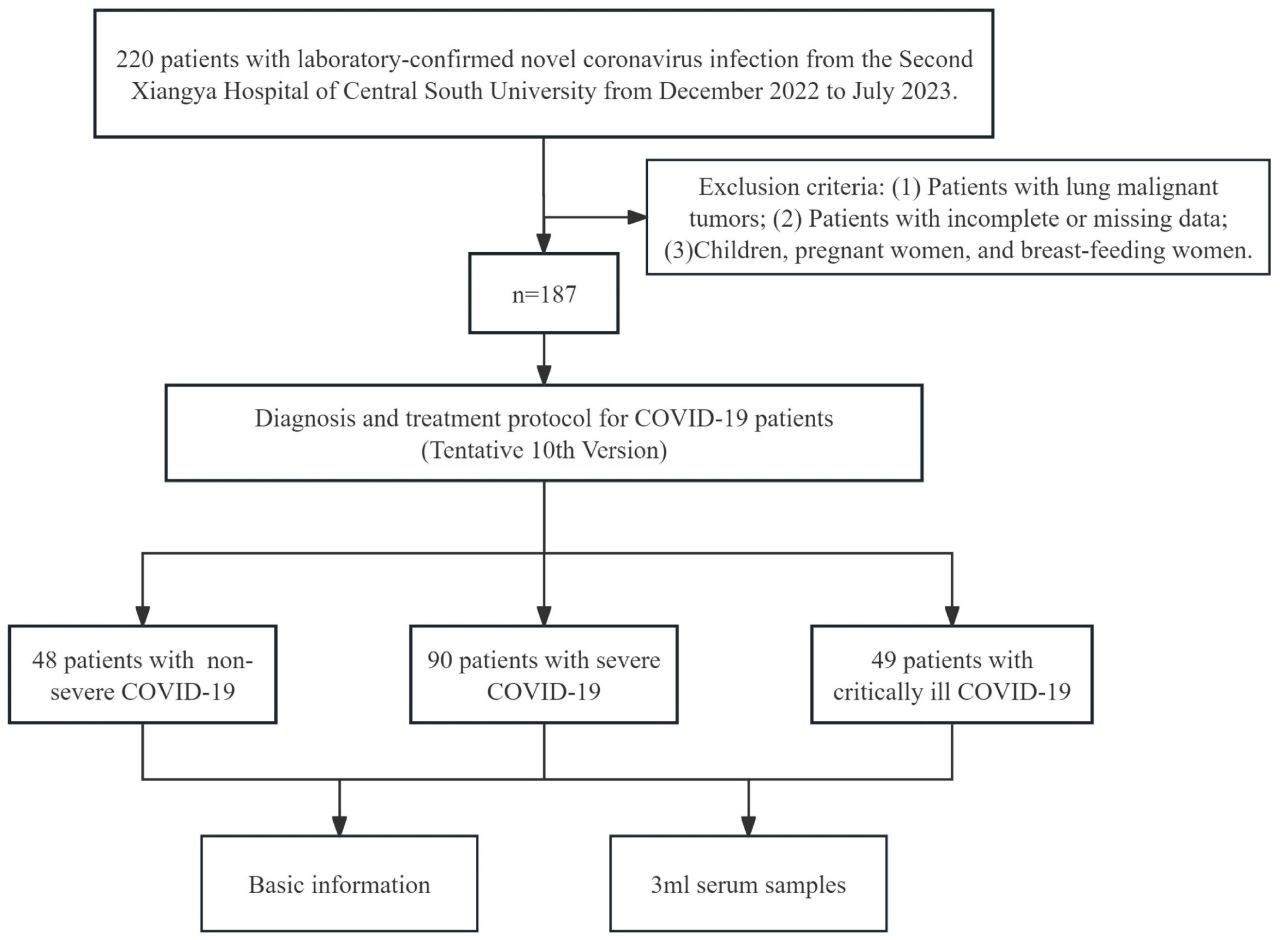
Selection of the study population.

The flow chart of patient enrollment is presented in Figure 1. The study was approved by the institutional ethics committee of the Second Xiangya Hospital of Central South University, and informed consent was required. All experiments were conducted in conformity with the applicable standards and regulations.

### Laboratory measurements

Using the ELISA kit, we determined the levels of OxLDL (Elabscience, Catalog#E-EL-H6021, China), 3-NT (Elabscience, Catalog#E-EL-0040c, China), TXB_2_ (Elabscience, Catalog#E-EL-H2191c, China) and 8-OHdG (Elabscience, Catalog#E-EL-0028c, China). A 50-fold dilution was required for all samples for the detection of OxLDL. For the detection of 3-NT, TXB_2,_ and 8-OHdG, the original fold was used, and an appropriate dilution of samples was needed when the concentration exceeded the maximum detection limit of the kit. The concentration was calculated by comparing the absorbance values from each sample to the standard curve.

The concentration of AOPP in serum was determined with AOPP Assay kits (Abbkine, Catalog#KTB1060, China). Samples required about a 5-fold dilution, which was achieved by the Extraction Buffer. The OD value of the product at 340 nm was determined and compared with the standard curve to confirm the content of AOPP in the samples.

The Hitachi 7600 automatic biochemical analyzer and the lipoprotein-associated phospholipase A_2_ assay (DiaSys Diagnostics Systems GmbH, Catalog#17181, Germany) kit were used to determine its activity. We calibrated it with calibrators and performed quality control, and then tested the sample.

### Sample size calculation

As previously described 21, 22, sample size calculation was performed using G * Power 3.1. And ANOVA: one-way (one independent variable) was chosen for the analysis. α err prob and power (1-β err prob) were set at 0.05 and 0.8, respectively. When the effect size f was equal to 0.25 and the number of groups was 3, the calculated results showed that the total sample size was 159.

### Statistical analysis

Statistical software SPSS 26.0 and MedCalc software were used to analyze the data. GraphPad Prism 9.5 was used for plotting. Categorical variables were expressed as frequencies (percentages). Continuous variables were expressed as mean ± standard deviation or median. The chi-square test was used for the comparison of percentages for categorical variables. Continuous variables with equal variance and normal distribution were statistically analyzed by ANOVA. Non-parametric tests were used to compare the data with an uneven variance or non-normal distribution. The correlation between oxidative stress markers and other parameters was determined by Spearman's test. The plot was generated using R software (v.4.2.2) package “corrplot” (v.0.92) 23 and “ggplot2” (v3.4.2) 24 through Hiplot Pro (https://hiplot.com.cn/), a comprehensive web service for biomedical data analysis and visualization. Univariate and multivariate ordered logistic regression were used to explore the correlation between disease severity and OS indicators. Lasso regression was used for variable filtering. Receiver operating characteristic (ROC) was used to analyze the diagnostic performance of the selected indexes by MedCalc software. The optimal cutoff value was determined by the Youden index, and the corresponding sensitivity and specificity were reported. All hypothesis testing was two-tailed, and *P* < 0.05 was considered to be statistically significant.

## Results

### Basic information

A total of 187 subjects were included in this study, including 48 patients with non-severe COVID-19, 90 patients with severe COVID-19, and 49 patients with critically ill COVID-19. The basic information for each group is shown in Table 1.

The age of patients in the severe group and critically ill group was higher than that in the non-severe group, and the critically ill patients were older than the severe patients. There were no statistical differences in the proportion of coronary heart disease, diabetes mellitus, chronic obstructive pulmonary disease (COPD), hypertension, hyperlipidemia, or malignancy. Critically ill COVID-19 patients had a higher proportion of males than non-severe COVID-19 patients.

As for laboratory indicators, there were no significant differences in ALT, TP, GLO, TBIL, DBIL, TBA, TG, and HDL-C (*P* > 0.05). The concentrations of ALB were significantly lower in the severe and critically ill groups compared with the non-severe group. CREA and CRP in the severe and critically ill groups were significantly higher than those in the non-severe group, and the critically ill group had the highest concentrations of CREA and CRP. Compared with the non-severe and severe groups, the TC and LDL-C were significantly decreased and the UREA was significantly increased in the critically ill group. In addition, AST and WBC levels were significantly higher in the critically ill patients than in the non-severe patients, but not significantly different from the severe patients.

### Oxidative stress indicators in the subjects

**Figure 2:**
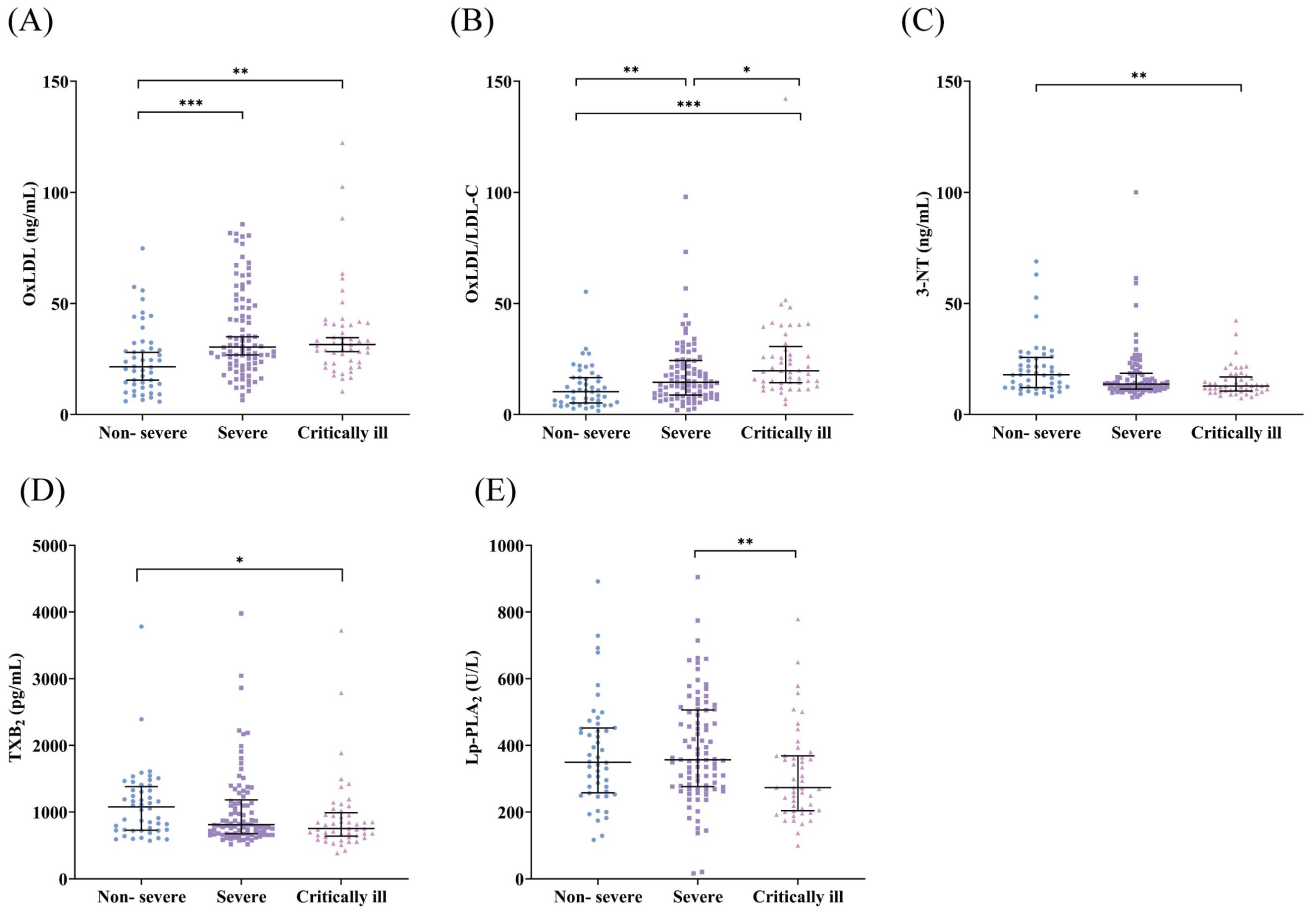
(A-E) Serum levels of OxLDL, OxLDL/LDL-C ratio, 3-NT, TXB_2_, and Lp-PLA_2_ in each group. OxLDL, oxidized low-density lipoprotein; LDL-C, low-density lipoprotein cholesterol; 3-NT, 3-nitrotyrosine; TXB_2_, thromboxane B_2_; Lp-PLA_2_, lipoprotein-associated phospholipase A_2_. *P < 0.05; **P < 0.01; ***P < 0.001.

As shown in Table 2 and Figure 2, compared with the non-severe group, OxLDL level, and OxLDL/LDL-C ratio were increased in severe COVID-19 patients and critically ill COVID-19 patients, and 3-NT and TXB_2_ concentrations were lower in critically ill COVID-19 patients. Critically ill COVID-19 patients had lower concentrations of Lp-PLA_2_ and a higher OxLDL/LDL-C ratio compared to severe COVID-19 patients. There were no significant differences in AOPP and 8-OHdG concentrations (*P* > 0.05).

### Correlation of oxidative stress indicators with CRP

**Figure 3:**
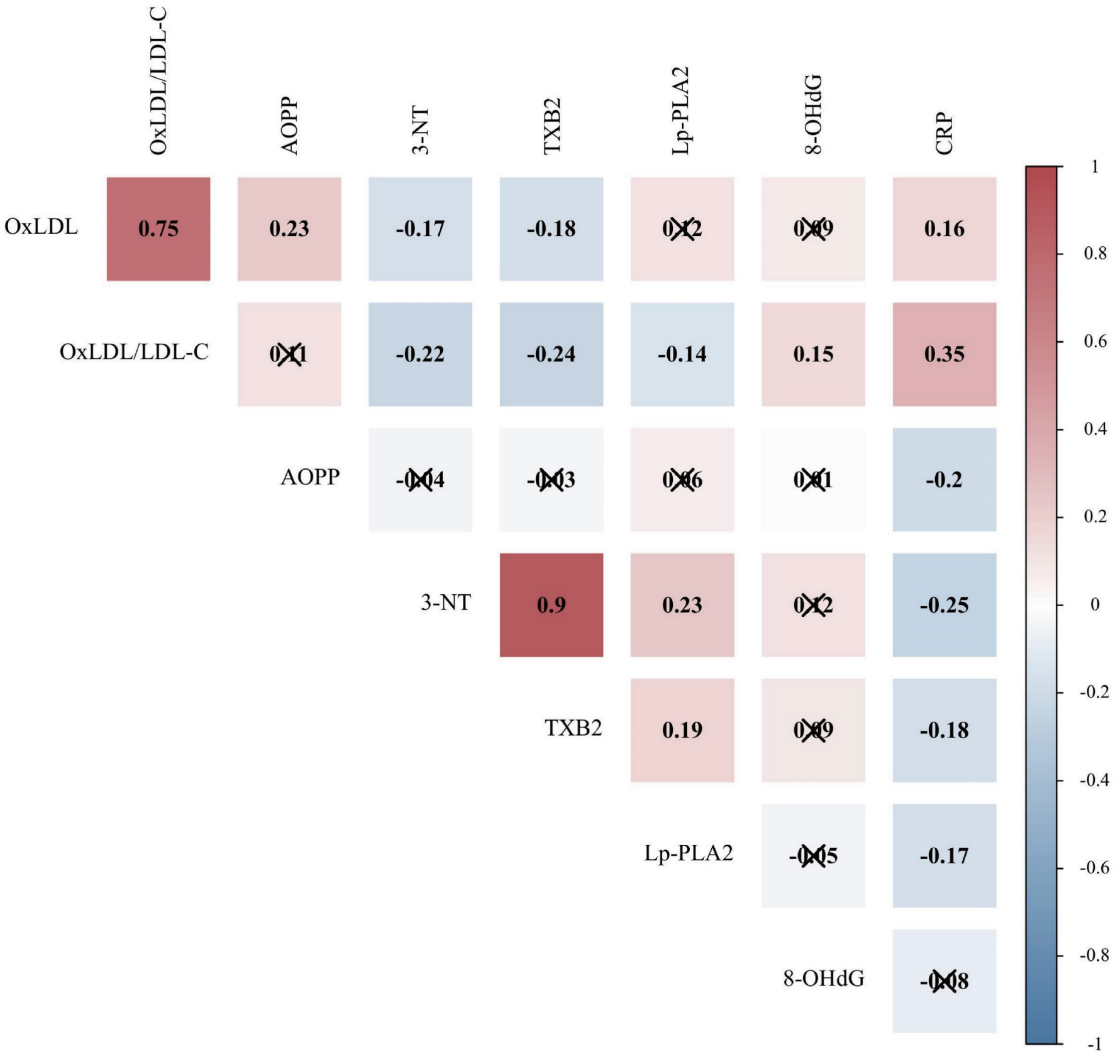
Correlation of oxidative stress indicators with CRP in patients with COVID-19. CRP, C-reactive protein; OxLDL, oxidized low-density lipoprotein; LDL-C, low-density lipoprotein cholesterol; AOPP, advanced oxidation protein products; 3-NT, 3-nitrotyrosine; TXB_2_, thromboxane B_2_; Lp-PLA_2_, lipoprotein-associated phospholipase A_2_; 8-OHdG, 8-hydroxydeoxyguanosine. Due to the non-normal distribution, the Spearman correlation matrix among different indicators is displayed, and the value represents the Spearman correlation coefficient. Positive correlations are shown in red and negative correlations are shown in blue (*P* < 0.05). Indicators that are not correlated are marked with “×” (*P* > 0.05).

Oxidative stress plays a synergistic role with inflammation in the progression of COVID-19. Therefore, the correlation between oxidative stress indicators and CRP was further evaluated. Spearman's correlation analysis was used to explore the relationships between OS indicators with statistical differences and CRP (n=187). It can be seen from the data in Figure 3 that CRP was associated with OxLDL, OxLDL/LDL-C ratio, AOPP, 3-NT, TXB_2_, and Lp-PLA_2_ (*P* < 0.05).

### Analysis of risk factors for severe/critically ill COVID-19

**Figure 4:**
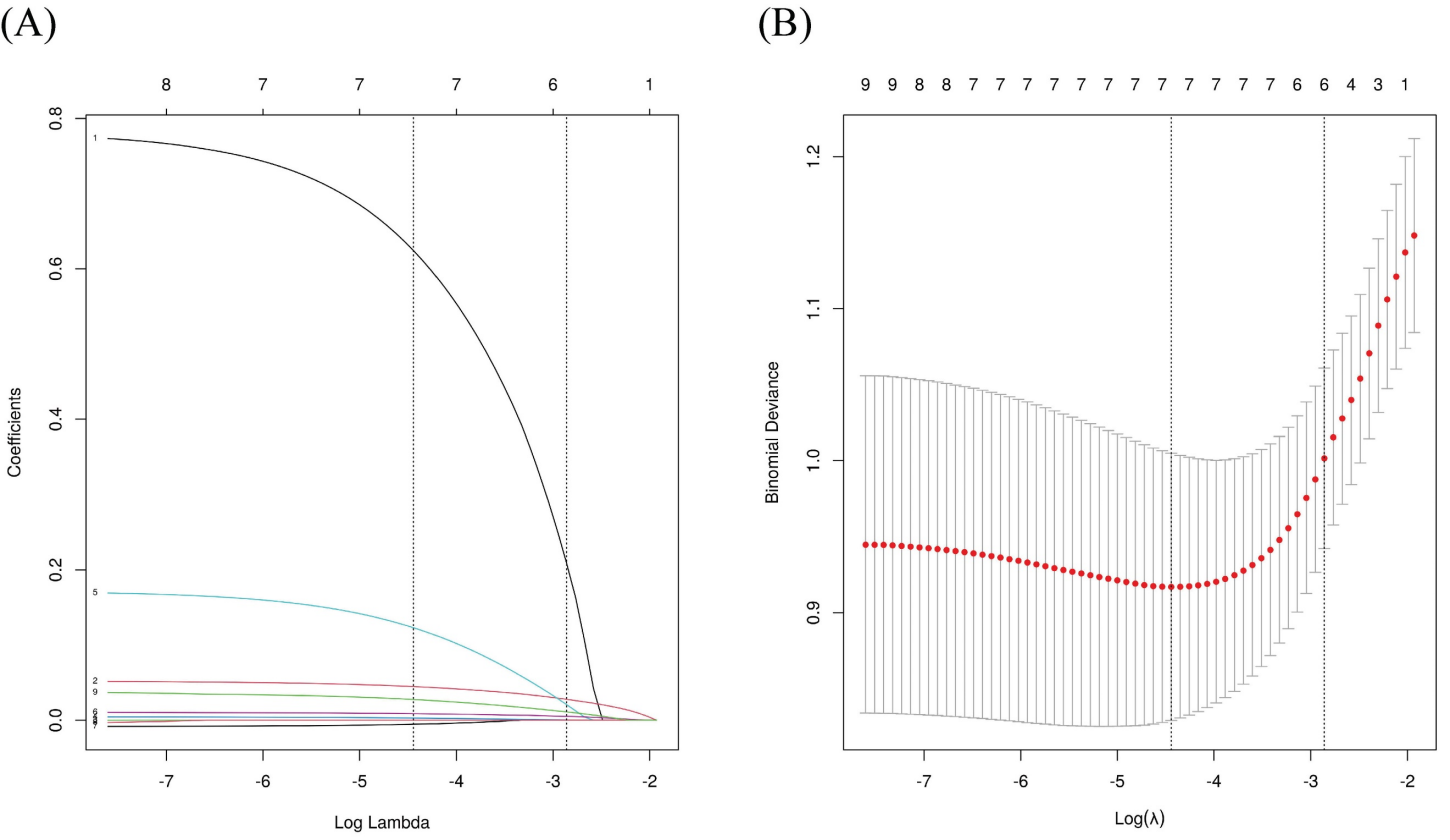
LASSO regression analysis for variable selection. (A) LASSO coefficient profiles; (B) Identification of the optimal penalization coefficient lambda (λ) in the LASSO model using 10-fold cross-validation. LASSO: least absolute shrinkage and selection operator.

We grouped severe and critically ill patients to further explore the risk factors for progression from non-severe to severe/critically ill COVID-19. Indicators with statistical differences between the groups in Table 1 and Table 2 were included in a univariate logistic regression analysis, revealing that age, male, ALB, CREA, WBC, CRP, OxLDL, OxLDL/LDL-C ratio, and 3-NT were associated with severe/critically ill infections. Variables with statistical differences in univariate logistic regression were included in LASSO regression to enable variable selection and prevent overfitting (Figure 4). Following LASSO regression analysis, gender, age, CREA, WBC, CRP, and OxLDL were included in the multivariate regression model. Further analysis indicated that OxLDL was an independent risk factor for severe/critical illness (*P* < 0.05), as shown in Table 3.

### ROC curve analysis of oxidative stress indicators in COVID-19

**Figure 5:**
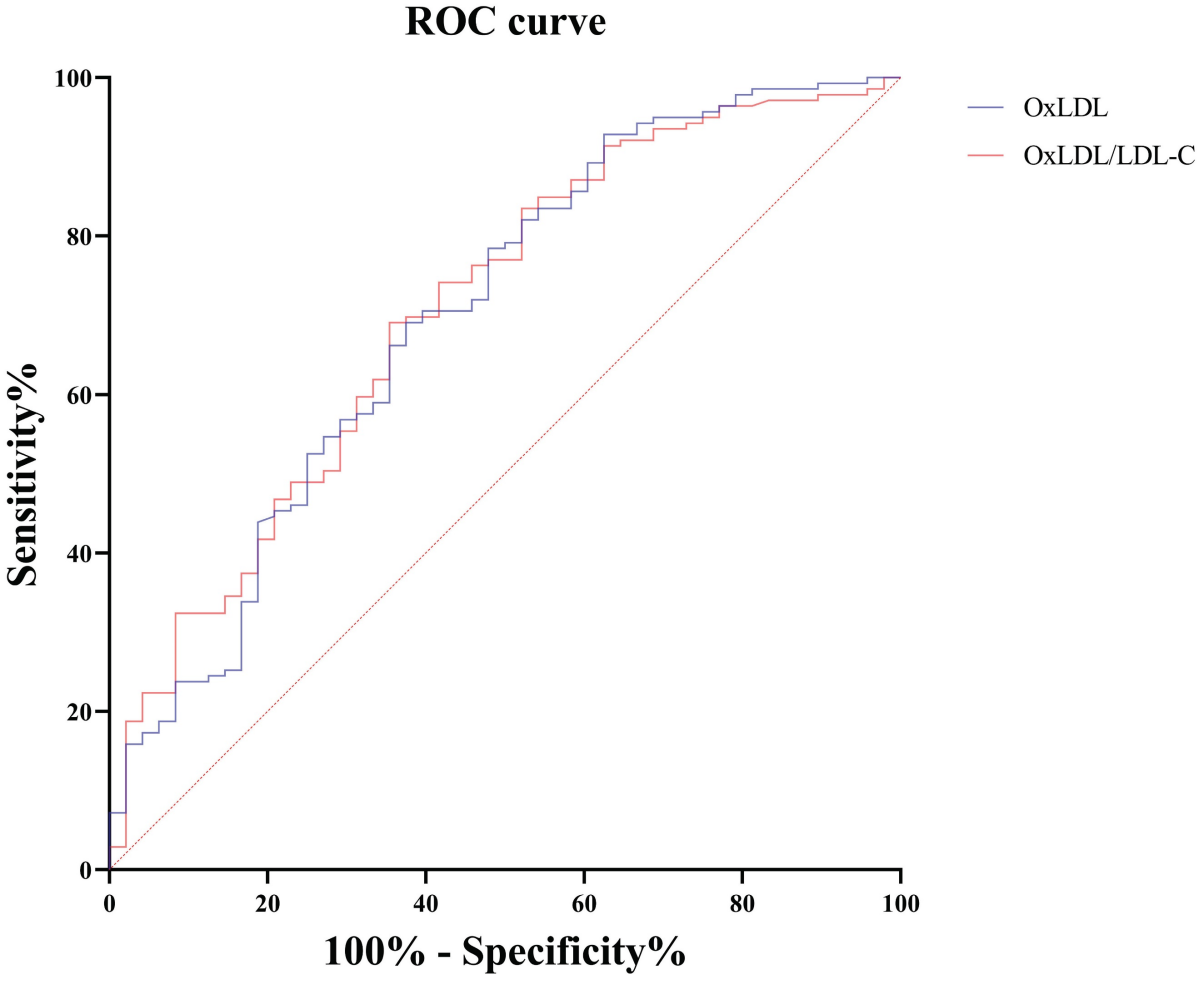
Receiver operating characteristic curves of serum OxLDL and OxLDL/LDL-C ratio for distinguishing severe/critically ill patients from non-severe patients. OxLDL, oxidized low-density lipoprotein; LDL-C, low-density lipoprotein cholesterol.

Among these markers of oxidative stress, only OxLDL was an independent risk factor for severe/critical illness, as shown in Table 3. Therefore, we investigated the role of OxLDL and OxLDL/LDL-C ratio in the identification of disease severity. Severe/critically ill patients were grouped to further explore their efficacy in distinguishing non-severe infections. The areas under the ROC curves (AUC) for 139 patients with severe/critically ill COVID-19 patients versus 48 patients with non-severe COVID-19 were 0.699 for OxLDL and 0.706 for the OxLDL/LDL-C ratio, as shown in Table 4 and Figure 5.

**Figure 6:**
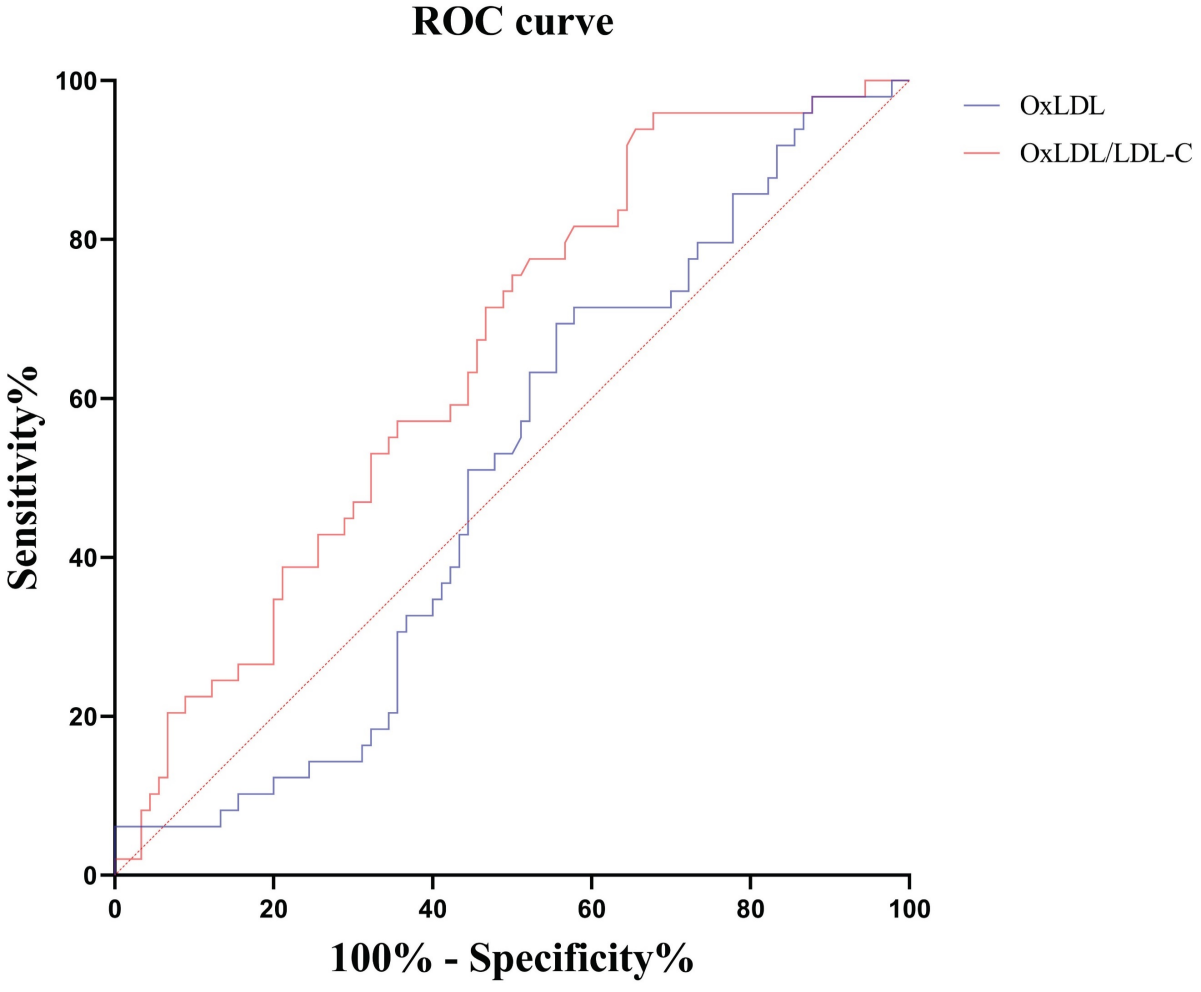
Receiver operating characteristic curves of serum OxLDL and OxLDL/LDL-C ratio for distinguishing critically ill patients from severe patients. OxLDL, oxidized low-density lipoprotein; LDL-C, low-density lipoprotein cholesterol.

We further explored the effect of these indicators in distinguishing severe and critically ill patients. The results showed that only the OxLDL/LDL-C ratio had a differential value. The area under the ROC curve (AUC) for 49 cases of critically ill COVID-19 versus 90 cases of severe COVID-19 was 0.653 for the OxLDL/LDL-C ratio (Table 5, Figure 6).

## Discussion

SARS-CoV-2 infection can lead to various clinical manifestations. Patients with severe and critically ill COVID-19 develop rapidly and experience a range of serious complications. Clinically, the prognosis for severe and critically ill COVID-19 patients is often worse than that of non-severe COVID-19 patients, with more severe clinical symptoms. It is well established that inflammation plays an important role in SARS-CoV-2 infection. Patients with more severe disease exhibit higher levels of CRP, indicating a greater degree of inflammation, as also reported by Trofin *et al.*^25^. Additionally, severe SARS-CoV-2 infection is associated with OS. Decreased activities of the antioxidant system have been observed in COVID-19 patients compared to healthy subjects 26, 27, suggesting alterations in oxidative damage substances in COVID-19 patients. In this report, we observed the changes in OS markers in patients of varying severity to identify feasible biomarkers for predicting severe disease.

In our study, it was found that the median age of patients with more severe infections was also higher and age was a risk factor for severe/critical illnesses. Therefore, older patients require closer monitoring for disease progression. Critically ill COVID-19 patients exhibited elevated levels of UREA and CREA, likely due to a higher incidence of impaired kidney function 28. Infectious diseases often lead to reduced ALB levels, and our results also observed that severe and critically ill patients had lower ALB levels than non-severe patients. Dysregulation of lipoprotein metabolism in patients with COVID-19 has been revealed, including changes in lipids and lipoproteins. We found that TC and LDL-C levels were lower in critically ill patients than in severe and non-severe patients, which supports evidence from previous observations 29, 30, indicating that critically ill patients present an altered lipid profile. Subsequently, we assessed the concentration of oxidative stress markers in COVID-19 patients from the perspective of oxidative damage to proteins, DNA, and lipids, and analyzed the role of these markers in severity assessment.

AOPP serves as an indicator of oxidative damage to proteins, representing oxidative modifications of plasma proteins such as albumin in response to ROS attacks. Additionally, 3-NT indicates high concentrations of nitrosative substances that lead to the destruction of natural protein structures. Our study found no statistical significance in AOPP levels between the non-severe, severe, and critically ill groups, consistent with previous studies 31, 32. However, we observed a significant decrease in 3-NT in the critically ill group and a downward trend in the severe group compared to the non-severe group. We hypothesized that the decline in 3-NT may be due to protein depletion or the body's protective mechanism after cellular protein damage.

8-OHdG is a marker of oxidative DNA damage caused by ROS. In our study, there was no statistical difference in 8-OHdG. Previously published studies on 8-OHdG changes have shown inconsistent results 33, 34, possibly due to differences in study population size and infection subtypes. More indicators are needed to reflect oxidative DNA damage accurately. OS, combined with cytokine storms, leads to platelet activation, blood clotting, and microvascular thrombosis by causing endothelial inflammation, endothelial cell dysfunction, and activation of the clotting cascade 7, 9. Lp-PLA_2_, an enzyme released from macrophages, mediates vascular inflammation by regulating lipid metabolism in the blood. The activity of Lp-PLA_2_ in patients with varying degrees of severity has not been well documented. Our results suggest that the activity of Lp-PLA_2_ is significantly lower in critically ill patients than in severe patients, with a decreasing trend compared to non-severe patients. This result is difficult to explain but may be related to lipid metabolism. The change in Lp-PLA_2_ is similar to that of TC and LDL-C, probably because Lp-PLA_2_ is mainly found in LDL-C 18. Therefore, Lp-PLA_2_ appears to reflect more abnormalities in lipid metabolism. TXB_2_ reflects platelet activation and pro-thrombotic activity. In our study, we found that compared with non-severe patients, critically ill patients had significantly lower TXB_2_ levels, and severe patients had a downward trend in TXB_2_ levels, which is consistent with the results obtained by RAVINDRAN *et al.* 20. It means that the production of TXB_2_ peaks at some point in response to viral infections. TXB_2_ was more consistent with changes in lipid mediator levels.

Lipid oxidation markers are also indicators of oxidative stress. Previous studies have also reported changes in other markers of lipid oxidative damage, including lipid peroxidation (LPO) and malondialdehyde (MDA). The level of LPO in COVID-19 patients was significantly higher than that in the control group. Moreover, LPO levels were higher in intubated/dead COVID-19 patients compared to mild patients 35. Compared with mild and moderate pneumonia, patients with severe pneumonia had higher levels of MDA 36. In addition, OS converts LDL-C to OxLDL, which plays a crucial role in initiating and promoting the inflammatory response and recruitment of white blood cells at the lesion site 37. Consequently, OxLDL is often considered a detrimental factor that causes bodily harm. At present, some studies have focused on changes in serum OxLDL in patients with COVID-19. In a prospective study, it was found that OxLDL levels in COVID-19 patients were higher than those in sex - and age-matched healthy controls, and the area under the ROC curve of OxLDL in differentiating COVID-19 from healthy people was 0.926, showing good efficacy 38. Similarly, in another report, blood OxLDL concentrations were found to be significantly higher in COVID-19 patients compared to controls 39. In our study, we found that serum OxLDL levels were significantly higher in severe or critically ill COVID-19 patients compared to non-severe COVID-19 patients. Previous studies have found that patients with severe pneumonia had higher OxLDL levels compared to patients with mild and moderate pneumonia, indicating that OxLDL concentrations were highest in patients with severe disease 36, which aligns with our results. Furthermore, OxLDL was identified as an independent risk factor for progression to severe or critical illness in this study. The risk of progression from non-severe to severe/critically ill COVID-19 increases with elevated OxLDL levels.

We hypothesize that severe and critically ill COVID-19 patients have higher lipid peroxidation levels. Lipids may be the main damaging substance in the severe infection phase, as lipid metabolism plays a key role in SARS-CoV-2 infection. However, the mechanism of OxLDL in SARS-CoV-2 infection has also not been revealed, and one hypothesis is that OxLDL may play a role in lung damage through macrophages. When OxLDL-trained macrophages encounter SARS-CoV-2 in the lung, it causes unregulated cytokine secretion, leading to alveolar damage 40. In addition, OxLDL was found to be associated with high inflammation in acute COVID-19. OxLDL may be another potential driver of inflammation in post-acute sequelae of SARS-CoV-2 (PASC) as it can activate the inflammasome through Toll-like receptor 4 and CD36 binding 41, 42. We observed that CRP was positively correlated with the OxLDL/LDL-C ratio and OxLDL in COVID-19 patients, suggesting consistency between lipoprotein oxidative damage and inflammation. The specific mechanism of OxLDL in the COVID-19 process remains to be further elucidated.

Most studies on COVID-19 have focused solely on OxLDL, often overlooking the OxLDL/LDL-C ratio. It is worth noting that the OxLDL/LDL-C ratio was significantly higher in severe or critically ill COVID-19 patients than in non-severe COVID-19 patients. One interesting finding is that there was a statistical difference in the OxLDL/LDL-C ratio between severe and critically ill cases, while no such difference was observed for OxLDL alone. One possible explanation for the decline in LDL-C in critically ill patients is its depletion due to ROS damage and conversion to OxLDL, which also suggests that the OxLDL/LDL-C ratio is worthy of attention.

Rapid identification of infections that may develop into severe and critical forms with simple indicators and active intervention is important for reducing the case fatality rate of COVID-19. Distinguishing between severe and critical infections also holds clinical value, as critically ill patients have a higher mortality rate and survivors may experience sequelae and long-term effects 43. To explore the diagnostic performance of OS indexes for COVID-19, further ROC curve analysis revealed that the OxLDL/LDL-C ratio is of differential value in distinguishing between non-severe and severe/critically ill COVID-19 patients, as well as between severe and critically ill COVID-19 patients. However, OxLDL only has a certain value in the identification of non-severe and severe/critically ill patients. Therefore, it is insufficient to observe the change in OxLDL, more attention should be paid to the ratio of OxLDL to LDL. According to these data, we can infer that the OxLDL/LDL-C ratio appears to be more indicative of disease severity than other markers of OS.

However, this study also has some shortcomings. Oxidative stress works collaboratively with inflammation and is involved in a variety of pathological processes, including COVID-19 8, 44. Meanwhile, this also means that similar to inflammatory indicators, oxidative stress indicators are non-specific and cannot be used as high-precision biomarkers. This study screened out the indexes of oxidative stress that are more significant for disease classification, but it is still a preliminary exploration. Therefore, more specific indicators are still to be discovered. Besides, this study was limited by the absence of disease control groups, preventing comparisons of oxidative stress (OS) levels between COVID-19 patients and those with other infectious diseases. The small sample size necessitates caution, as some results showed trends without statistical significance. Additionally, this study did not dynamically monitor the levels of the aforementioned indicators after admission. Consequently, it remains unclear how OS measures change over time in the same patient or when these measures peak. In addition, some patients had taken drugs for basic diseases and symptomatic drugs for COVID-19. However, the study did not explore which medications the patients were taking before blood collection, and the effects of these drugs on the results were not revealed in this paper. Considerably more work will need to be done to determine the predictive value of OS indicators for severe disease.

In summary, this study shows that patients with severe and critically ill COVID-19 exhibit higher levels of oxidative damage to lipoproteins. Additionally, CRP is correlated with OxLDL and the OxLDL/LDL-C ratio. OxLDL and the OxLDL/LDL-C ratio have significant value in distinguishing between non-severe and severe/critically ill COVID-19. Furthermore, the OxLDL/LDL-C ratio can also differentiate between severe and critically ill patients. Therefore, OxLDL and the OxLDL/LDL-C ratio can be used as indicators to assess the severity of COVID-19 patients.

## Figures and Tables

**Table 1:** Baseline characteristics of the subjects.

Demographics	Non-severe (n=48)	Severe (n=90)	Critically ill(n=49)	*P* value
Age (years)	64(55, 79)	74(67, 83)^ *^	82(73, 87)^ *#^	<0.001
Male, n (%)	26(54.2%)	63(70.0%)	42(85.7%) ^*^	0.003
Coronary heart disease, n (%)	12(25.0%)	22(24.4%)	21(42.9%)	0.055
Diabetes mellitus, n (%)	17(35.4%)	34(37.8%)	23(46.9%)	0.454
COPD, n (%)	2(4.2%)	13(14.4%)	5(10.2%)	0.176
Hypertension, n (%)	26(54.2%)	61(67.8%)	36(73.5%)	0.115
Hyperlipidemia, n (%)	7(14.6%)	6(6.7%)	3(6.1%)	0.237
Malignancy, n (%)	2(4.2%)	7(7.8%)	4(8.2%)	0.757
Clinical and biological parameters				
ALT (U/L)	24.05(14.88, 35.23)	28.00(16.85, 38.58)	23.10(14.30, 42.80)	0.649
AST (U/L)	25.60(19.23, 41.28)	29.85(22.05, 45.53)	37.40(26.40, 61.45) ^*^	0.007
TP (g/L)	62.58±7.52	60.66±6.77	61.00±7.23	0.306
ALB (g/L)	34.49±5.42	32.45±5.15 ^*^	31.47±4.71 ^*^	0.013
GLO (g/L)	28.09±5.36	28.21±4.87	29.53±6.37	0.319
TBIL (μmol/L)	9.50(6.30, 12.00)	9.80(7.03, 12.03)	9.50(5.85, 13.85)	0.860
DBIL (μmol/L)	3.45(2.43, 4.48)	3.80(2.70, 5.40)	3.70(2.80, 6.10)	0.266
TBA (μmol/L)	4.15(2.43, 6.50)	4.90(3.10, 8.40)	4.90(3.05, 9.85)	0.376
UREA (mmol/L)	5.94(4.50, 9.55)	7.20(5.06, 11.68)	11.95(6.95, 19.92) ^*#^	<0.001
CREA(μmol/L)	69.00(56.75, 97.78)	80.55(67.30, 137.53)^ *^	118.00(84.55, 188.40)^ *#^	<0.001
TG (mmol/L)	1.32(0.92, 2.11)	1.35(1.01, 1.76)	1.36(1.10, 1.89)	0.980
TC (mmol/L)	4.12(3.19, 5.00)	3.91(3.25, 4.61)	3.39(2.57, 3.89)^ *#^	<0.001
HDL-C (mmol/L)	0.91(0.73, 1.15)	0.92(0.75, 1.07)	0.95(0.75, 1.18)	0.905
LDL-C (mmol/L)	2.40±1.06	2.41±0.96	1.68±0.69 ^*#^	<0.001
WBC (×10^9^/L)	6.09(4.66, 7.54)	6.83(4.91, 9.78)	7.29(5.98, 10.83)^ *^	0.007
CRP (mg/L)	30.10(9.89, 81.20)	77.90(22.93, 114.50)^ *^	107.03(62.85, 182.00)^ *#^	<0.001

Notes: COPD, chronic obstructive pulmonary disease; ALT, alanine transaminase; AST, aspartate transaminase; TP, total protein; ALB, albumin; GLO, globular protein; TBIL, total bilirubin; DBIL, direct bilirubin; TBA, total bile acid; CREA, creatinine; TG, triglyceride; TC, total cholesterol; HDL-C, high-density lipoprotein cholesterol; LDL-C, low-density lipoprotein cholesterol; WBC, white blood cell; CRP, C-reactive protein. ^*^Compared with non-severe group, *P*<0.05; ^#^Compared with severe group, *P*<0.05.

**Table 2:** Levels of oxidative stress indicators in non-severe and severe COVID-19 patients.

	Non-severe (n=48)	Severe (n=90)	Critically ill(n=49)	*P* value
OxLDL (ng/mL)	21.50(13.03, 31.59)	30.43(22.57, 49.57) ^*^	31.54(23.47, 40.81) ^*^	<0.001
OxLDL/LDL-C	10.30(5.23, 16.59)	14.49(8.84, 24.26) ^*^	19.64(14.35, 30.64) ^*#^	<0.001
AOPP (μmol/L)	263.95(157.11, 390.52)	270.75(176.48, 350.51)	277.89(155.02, 378.55)	0.786
3-NT (ng/mL)	17.90(12.13, 25.72)	13.69(11.45, 18.57)	12.83(10.62, 16.92)^ *^	0.004
TXB_2_ (pg/mL)	1075.87(726.50, 1380.74)	808.58(673.04, 1182.06)	752.20(637.48, 988.15)^ *^	0.017
Lp-PLA_2_ (U/L)	349.20(257.48, 451.78)	356.45(275.98, 506.00)	273.10(203.90, 368.70) ^#^	0.006
8-OHdG (ng/mL)	52.52(36.17, 93.89)	61.55(36.95, 88.47)	54.26(39.87, 112.95)	0.823

Notes: OxLDL, oxidized low-density lipoprotein; LDL-C, low-density lipoprotein cholesterol; AOPP, advanced oxidation protein products; 3-NT, 3-nitrotyrosine; TXB_2_, thromboxane B_2_; Lp-PLA_2_, lipoprotein-associated phospholipase A_2_; 8-OHdG, 8-hydroxydeoxyguanosine. *Compared with non-severe group, *P*<0.05; #Compared with severe group, *P*<0.05.

**Table 3:** Univariate and multivariate Logistic regression analysis of risk factors for severe/critically ill COVID-19.

Variables	Univariate Logistic regression analysis			Multivariate Logistic regression analysis	
	OR (95% CI)	*P* value		OR (95% CI)	*P* value
Age (years)	1.060(1.032, 1.089)	<0.001		1.051(1.020, 1.082)	0.001
Gender (male)	2.613(1.315, 5.194)	0.006		2.493(1.111, 5.594)	0.027
AST (U/L)	1.014(0.998, 1.030)	0.091			
ALB (g/L)	0.915(0.857, 0.976)	0.007			
UREA (mmol/L)	0.996(0.989, 1.004)	0.335			
CREA (μmol/L)	1.006(1.001, 1.012)	0.030		—	0.051
TC (mmol/L)	0.818(0.622, 1.075)	0.149			
LDL-C (mmol/L)	0.777(0.559, 1.079)	0.132			
WBC (×109/L)	1.186(1.048, 1.342)	0.007		1.159(1.001, 1.342)	0.049
CRP (mg/L)	1.015(1.007, 1.022)	<0.001		1.011(1.003, 1.020)	0.006
OxLDL (ng/mL)	1.046(1.020, 1.073)	0.001		1.036(1.009, 1.064)	0.009
OxLDL/LDL-C	1.074(1.031, 1.118)	0.001			
3-NT (ng/mL)	0.972(0.946, 0.998)	0.034			
TXB_2_ (pg/mL)	1.000(0.999, 1.000)	0.226			
Lp-PLA_2_ (U/L)	0.999(0.997,1.002)	0.592			

Notes: Univariate logistic regression analysis was conducted to explore the risk/protective factors of COVID-19, and further multivariate logistic regression analysis was conducted to explore the independent risk/protective factors of the disease after adjusting for other variables. OR, odds ratio; CI, confidence interval. AST, aspartate transaminase; ALB, albumin; CREA, creatinine; TC, total cholesterol; LDL-C, low-density lipoprotein cholesterol; WBC, white blood cell; CRP, C-reactive protein. OxLDL, oxidized low-density lipoprotein; 3-NT, 3-nitrotyrosine; TXB_2_, thromboxane B_2_; Lp-PLA_2_, lipoprotein-associated phospholipase A_2_.

**Table 4:** ROC curve analysis of oxidative stress indexes in distinguishing severe/critically ill patients from non-severe patients.

Indicators	n	AUC	95%CI	*P* value	Threshold	Sensitivity, %(95%CI)	Specificity, %(95%CI)	Youden index
OxLDL (ng/mL)	187	0.699	0.627, 0.763	<0.001	25.59	69.1(60.7, 76.6)	62.5(47.4, 76.0)	0.316
OxLDL/LDL-C	187	0.706	0.635, 0.770	<0.001	12.42	69.1(60.7, 76.6)	64.6(49.5, 77.8)	0.337

Notes: CI, confidence interval. OxLDL, oxidized low-density lipoprotein; LDL-C, low-density lipoprotein cholesterol.

**Table 5:** ROC curve analysis of oxidative stress indexes in distinguishing critically ill patients from severe patients.

Indicators	n	AUC	95%CI	*P* value	Threshold	Sensitivity, %(95%CI)	Specificity, %(95%CI)	Youden index
OxLDL (ng/mL)	139	0.504	0.418, 0.590	0.940	—	—	—	—
OxLDL/LDL-C	139	0.653	0.568, 0.732	0.001	10.73	93.9(83.1, 98.7)	34.4(24.7, 45.2)	0.283

Notes: CI, confidence interval. OxLDL, oxidized low-density lipoprotein; LDL-C, low-density lipoprotein cholesterol.
